# prot4EST: Translating Expressed Sequence Tags from neglected genomes

**DOI:** 10.1186/1471-2105-5-187

**Published:** 2004-11-30

**Authors:** James D Wasmuth, Mark L Blaxter

**Affiliations:** 1 Institute of Evolutionary Biology, School of Biological Sciences, University of Edinburgh, EH9 3JT, UK

## Abstract

**Background:**

The genomes of an increasing number of species are being investigated through generation of expressed sequence tags (ESTs). However, ESTs are prone to sequencing errors and typically define incomplete transcripts, making downstream annotation difficult. Annotation would be greatly improved with robust polypeptide translations. Many current solutions for EST translation require a large number of full-length gene sequences for training purposes, a resource that is not available for the majority of EST projects.

**Results:**

As part of our ongoing EST programs investigating these "neglected" genomes, we have developed a polypeptide prediction pipeline, prot4EST. It incorporates freely available software to produce final translations that are more accurate than those derived from any single method. We show that this integrated approach goes a long way to overcoming the deficit in training data.

**Conclusions:**

prot4EST provides a portable EST translation solution and can be usefully applied to >95% of EST projects to improve downstream annotation. It is freely available from .

## Background

### The need for more sequence

Complete genome sequencing is a major investment and is unlikely to be applied to the vast majority of organisms, whatever their importance in terms of evolution, health or ecology. Complete genome sequences are available for only a few eukaryote genomes, most of which are model organisms. The focus of eukaryote genome sequencing has been on a restricted subset of known diversity, with, for example, nearly half of the completed or draft stage genomes being from vertebrates. While Arthropoda and Nematoda have two completed genomes each, with a dozen others in progress, compared to predicted diversity (over a million species each) current genome sequencing illuminates only small parts of even these phyla. The disparity between sequence data and motivation for biological study is significant. Allied to this bias in genome sequence is a bias in functional annotation for the derived proteomes: a vertebrate gene is more likely to have been assigned a function due to the focus of biomedical research on humans and closely related model species such as mouse [1].

Shotgun sample sequencing of additional genomes through expressed sequence tags (EST) or genome survey sequences (GSS) has proved to be a cost-effective and rapid method of identifying a significant proportion of the genes of a target organism. Thus many genome initiatives on non-traditional model organisms have utilised EST and GSS strategies to gain an insight into "wild" biology. An EST strategy does not yield sequence for all of the expressed genes of an organism, because some genes may not be expressed under the conditions sampled, and others may be expressed at very low levels and missed through the random sampling that underlies the strategy. However the creation of EST libraries from a range of conditions, such as different developmental stages or environmental exposures, promotes a closer examination of the biology of these species.

**Figure 1:**
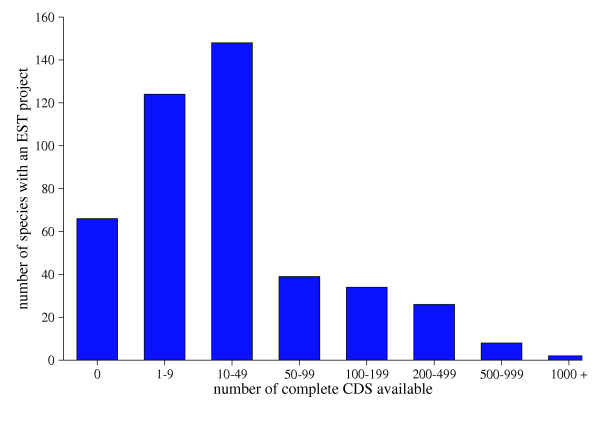
**The training set deficit for EST projects. **Around 85% of species with representation in dbEST (>100 ESTs) have less than 100 complete CDS entries in the EMBL database. These species comprise ~45% of all ESTs. Sixty-six species, with 246263 dbEST sequences, have no full-length CDS. Source: dbEST and EMBL database (July 2004).

The well documented phylogenetic sequence deficit [2] has led us to coin the term "neglected genomes". Currently many groups are sequencing ESTs from their chosen species to perform studies in a wide-range of disciplines, from comparative ecotoxicology [3] to high-throughput detection of sequence polymorphisms [4,5]. The contribution of EST projects for neglected but biologically relevant organisms is highlighted in Figure 1. As with all sequence data, obtaining high quality annotation requires prior information and is labour intensive. The "partial genome" information that results from EST datasets presents special problems for annotation, and we are developing tools for this task.

### The need for high quality translation

The PartiGene software suite [6] simplifies the analysis of partial genomes. ESTs are clustered into putative genes and consensuses determined. All the data is stored in a relational database, allowing it to be searched easily. While preliminary annotation based on BLAST analysis of nucleotide sequence can be performed, more robust methods are needed to allow high-quality analysis. The error-prone nature of ESTs makes application of most annotation tools difficult. To improve annotation, and facilitate further exploitation, a crucial step is the robust translation of the EST or consensus to yield predicted polypeptides. The polypeptide sequences present a better template for almost all annotation, including InterPro [7] and Pfam [8], as well as the construction of more accurate multiple sequence alignments, and the creation of protein-mass fingerprint libraries for proteomics exploitation. High quality polypeptide predictions can be applied to functional annotation and post-genomic study in a similar way to those available for completed genomes.

### Translating Expressed Sequence Tags

Prediction of the correct polypeptide from ESTs is not trivial:

1. The inherent low quality of EST sequences may result in shifts in the reading frame (missing or inserted bases) or ambiguous bases. These errors impede the correct recognition of coding regions. The initiation site may be lost, or an erroneous stop codon introduced to the putative translation.

2. ESTs are often partial segments of a mRNA, and as most cloning technology biases representation to the internal parts of genes, the initiation methionine codon may be missed. This is a problem for some of the *de novo *programs which use the initiation methionine to identify the coding region (described below).

Sequence quality can be improved by clustering the sequences based on identity. For each cluster a consensus can be determined [9]. This approach, however, will not address the whole problem as poor quality EST sequences may not yield high quality consensuses and for smaller volume projects, most genes have a single EST representative. Therefore additional methods must be applied to provide accurate polypeptide predictions.

### Similarity-based methods

A robust method to determine the correct encoded polypeptide is to map a nucleotide sequence onto a known protein. This concept is the basis for BLASTX [10], FASTX [11] and ProtEST [12]. BLASTX and FASTX use the six frame translation of a nucleotide sequence to seed a search of a protein database. The alignments generated for each significant hit provide an accurately translated region of the EST. BLASTX is extremely rapid, but the presence of a frameshift terminates each individual local alignment, ending the polypeptide prematurely. FASTX is able to identify possible frameshifts, but its dynamic programming approach is significantly slower than BLASTX. These methods require that the nucleotide sequence shares detectable similarity with a protein in the selected database. Many genes from both well studied and neglected genomes do not share detectable similarity to other known proteins. For example, the latest analysis of the *Caenorhabditis elegans *proteome shows that only ~50% of the 22000 predictions contain Pfam-annotated protein domains [8,13], and 40% share no significant similarity with non-nematode proteins in the SwissProt/trEMBL database [14]. This feature is not unique to the phylum Nematoda, and is likely perhaps to be more extreme for neglected genomes, given the phylogenetic bias of most protein databases.

ProtEST uses a slightly different similarity-based approach [12]. A protein sequence is compared to an EST database. phrap [9] is used to construct a consensus sequence from the ESTs found to have significant similarity. These consensuses are then compared to the original sequence using ESTWISE (E. Birney, unpublished [15]) giving a maximum likelihood position for possible frameshifts. The system is accurate but is not readily adaptable to the high-throughput approach necessary when dealing with very large numbers of ESTs. More crucially, an EST that does not show significant similarity to a known protein is not translated.

### 'de novo' predictions

To overcome the reliance upon sequence similarity, *de novo *approaches based on recognition of potential coding regions within poor quality sequences, reconstruction of the coding regions in their correct frame, and discrimination between ESTs with coding potential and those derived from non-coding regions have been developed [16-18].

DIANA-EST [16], combines three Artificial Neural Networks (ANN), developed to identify the transcription initiation site and the coding region with potential frameshifts. ESTScan2 [18] combines three hidden Markov models trained to be error tolerant in their representations of mRNA structure (modelling the 5' and 3' untranslated regions, initiation methionine and coding region). DECODER [17] uses an essentially rule-based method for identifying possible insertions and deletions in the nucleotide sequence, as well as the most likely initiation site, and was developed for complete cDNA sequence translation.

Each of these methods has different strengths in their attempt to identify the precise coding region; all require prior data to train their models. Published descriptions of their utility are based on training with human full length coding sequences (mRNAs), and thus tens of thousands of training sequences (many million coding nucleotides) were used to achieve optimum results. As stressed above, this amount of prior data is not available for the vast majority of EST project species (Figure 1).

### New solution – prot4EST

Prior to this project, nematode ESTs available through NEMBASE [19] had been translated using DECODER, as a preliminary study had suggested that it outperformed the other available methods (DIANA-EST and ESTScan1 [20]) (Parkinson pers. com.). 7388 out of the 40000 resulting predicted polypeptides were likely to be poorly translated (<30 amino acids), and we suspected many more contained errors. This motivated the creation of a solution using several methods to enhance the quality of the polypeptide predictions, exploiting their strengths while recognising their short-comings. prot4EST is an EST translation pipeline, written in Perl, with a user-friendly interface, that links some of these described methods together. It carries out retrieval and formatting of files from online databases for the user. It has been designed to be used as a stand-alone tool, or as an integral part of the PartiGene process [6].

## Implementation

### DECODER

The DECODER program [17] was developed to define start codons and open reading frames in full-length cDNA sequences. It exploits the quality scores for the sequence produced from base-calling software, such as phred [21,22], and additional text-based information to identify all possible coding regions. In regions of low sequence quality up to 2 nucleotides are removed or inserted, representing possible frameshifts. A likelihood score is calculated for each possible coding sequence (CDS), and the one with the lowest score is chosen as the correct CDS. The score is computed from the probability of generating a random sequence with a better Kozak consensus (the nucleotide sequence surrounding the initiation codon of a eukaryotic mRNA), ATG position and codon usage. DECODER requires a codon bias table, which is used to determine the putative coding regions optimal codon usage. A penalty term limits the number of insertions/deletions in the corrected CDS.

### ESTScan2.0

Hidden Markov models (HMM) can represent known sequence composition in a probabilistic manner [23]. This has been exploited recently in applications to find genes in genomic sequence [24,25], predict domain composition in protein sequences [26], and align multiple sequences [27]. ESTScan [18] exploits the predictive power of Hidden Markov models by combining three models:

1. Modeling mRNA structure: ESTScan separates the probable CDS from the untranslated regions (UTRs). The core of the coding sequence is represented by a 3-periodic inhomogeneous hidden Markov model. Flanking this core model are start and stop profiles for the codons observed at these positions. The profiles for untranslated regions flank the start and stop states.

2. Error tolerance: ESTScan allows insertions and deletions (indels) in the EST sequence. For example, if it is more probable that a particular nucleotide is the result of an insertion event then it is omitted from the 'corrected' sequence. Conversely, if the HMM probability scores suggest that a nucleotide has been deleted then the model inserts an X into the 'corrected' sequence to denote this prediction.

3. EST structure: ESTScan recognises that the EST may be composed of a combination of 5' UTR, CDS and 3' UTR.

ESTScan's hidden Markov models are trained using complete CDS entries from either the EMBL or RefSeq databases. Scripts included with the distribution parse the data files, extracting the necessary sequence information to produce the model files. The major issue considered at this point is redundancy. If the training data is internally redundant then the resultant model will be fully successful only in finding what is known and will have reduced power in detecting novel transcripts. Default parameters were used in ESTScan for building the HMM and in predicting polypeptides.

### HSP tiling

The BLASTX program [10] allows a nucleotide sequence to be searched against a protein database. The nucleotide query is translated in all six frames and these are used as the query sequences for a BLASTP search. High scoring segment pairs (HSP) are identified that maximise a bit score derived from an amino acid similarity matrix. If a single indel occurs in the nucleotide sequence, causing a frameshift, the HSP is either terminated at this position or continues out of frame. Downstream of this frameshift the query sequence may be long enough to result in another significant HSP to the same protein sequence, this time in a different frame. Simple extraction of the best BLAST HSP will miss such features. prot4EST implements a rule-based method that considers all the HSPs for a match to a database sequence and considers whether a frameshift can be identified. Where a frameshift is identified the HSPs are joined. Where two HSPs overlap the sequence with the better bit score is used.

### The prot4EST pipeline

**Figure 2:**
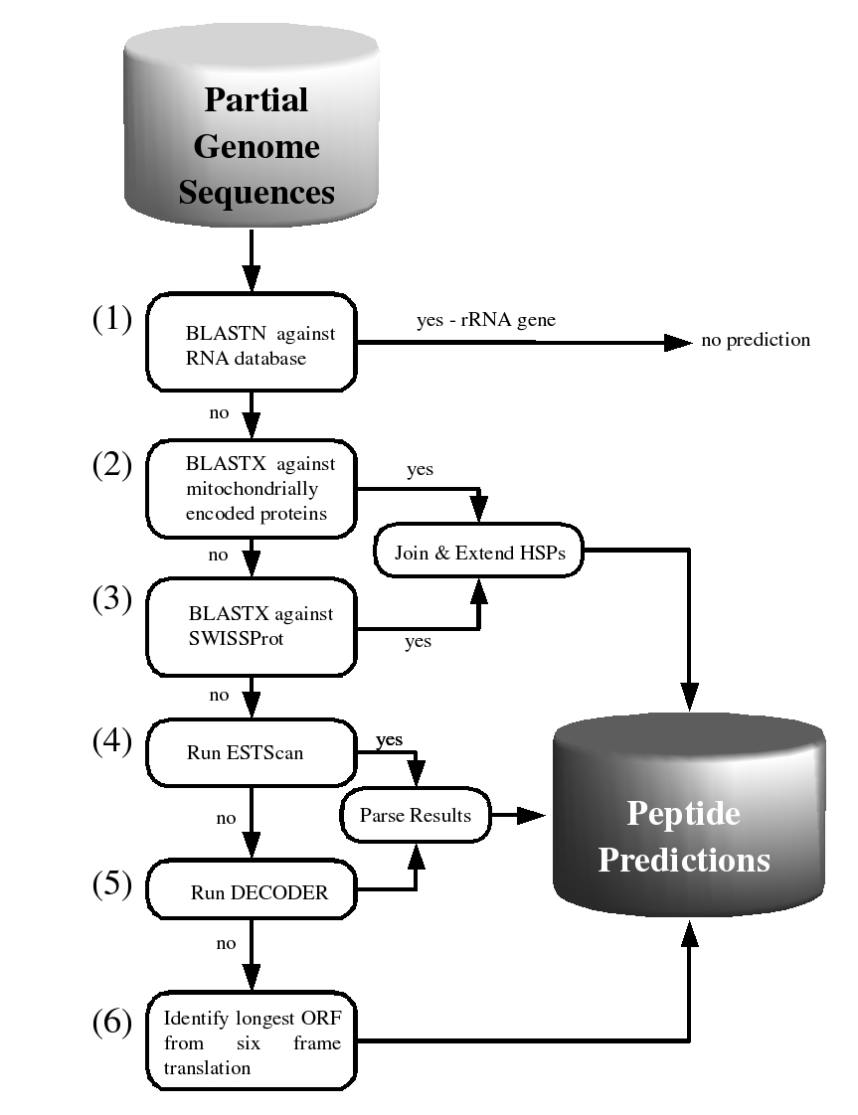
The prot4EST pipeline.

prot4EST is an integrated pipeline utilising freely available software in a tiered, rule-based system (Figure 2).

#### Tier 1: Identification of ribosomal RNA (rRNA) genes

The protein databases contain (probably spurious) translations of ribosomal RNA genes and gene fragments, and thus it is important to identify and remove putative rRNA derived sequences before further processing. A BLASTN search is performed against a database of rRNA sequences obtained from the Ribosomal Database II (Table 1; [28]). A BLAST expect value cutoff of e-65 is used to identify matches. The cutoff is a conservative one to reduce the number of false positives. Those nucleotide sequences with significant matches are annotated as rRNA genes and take no further part in the translation process.

#### Tiers 2 and 3: Similarity search

The second and third stages are similar. First a BLASTX search is performed against proteins encoded by mitochondrial genomes. The mitochondrial protein database is obtained from the NCBI ftp site (Table 1). Any sequences with significant hits (cutoff e-8) are annotated as mitochondrion-encoded genes for the remainder of the process, and the relevant mitochondrial genetic code is used for translation. Sequences that do not have significant similarity to mitochondrial proteins are compared using BLASTX to the SwissProt database [14]. Sequences that yield no significant similarity are moved onto tier 4 of the process.

For those sequences that show significant similarity to a protein sequence from either database a HSP tile path is constructed. prot4EST then considers whether the nascent translation can be extended at either end in the same reading frame.

#### Tier 4: ESTScan prediction

The hidden Markov models used by ESTScan to identify the coding region are constructed from EMBL format files for complete CDS using scripts supplied with the package. Preprocessing is integrated within prot4EST, including the downloading of the EMBL files. A pair of length threshold criteria are applied to each putative polypeptide before it is accepted. The open reading frame must be at least 30 codons in length, and cover at least 10% of the input sequence. Polypeptides that satisfy these criteria undergo the extension process described above, sequences that fail any of the criteria are passed onto the next tier. The extension process is carried out on those sequences that exceed the thresholds.

#### Tier 5: DECODER prediction

The DECODER program is used to predict CDS and thus polypeptide translations for the remaining nucleotide sequences. For each sequence a quality file in phrap format is required. When a quality file is unavailable a file with quality values of 15 is generated for each sequence. The codon usage table required by DECODER can be specified by the user or downloaded from CUTG, the codon usage table database [29]. By default DECODER only processes the forward strand of each sequence, and therefore the reverse complement of each sequence is taken and processed through DECODER. Two putative polypeptides are generated for each nucleotide sequence. The longer polypeptide is selected as the more probable translation. The polypeptide predictions are checked using the same length threshold criteria as for ESTScan (above).

#### Tier 6: Longest ORF

This last attempt to provide a putative polypeptide translation determines the longest string of amino acids uninterrupted by stop codons from a six-frame translation of the sequence. If a methionine is present in this string it is flagged as a potential initiation site.

## Output

The primary output from prot4EST consists of the putative polypeptides in FASTA format, complemented with files containing information describing the translated sequences. This information includes:

position of the translation with respect to the nucleotide sequence, the genetic code used for translation,

position and BLAST statistics of HSPs used in the tile path.

All this additional information is stored in two CSV format files, permitting parsing and simple insertion into a database.

## Speed

This is highly dependent upon the composition and size of the dataset. As a guide, each prot4EST run carried out in the benchmarking (below), took less than an hour for a 2316-sequence input with an Athlon 1400 Mhz processor. The BLASTX searches were carried out separately and used as input to prot4EST (for details see the userguide, availabile from the program web page).

## Benchmarking EST translation methods

We benchmarked five translation methods to test their relative performance. DECODER is designed to consider only the forward strand of the nucleotide sequence, as it was originally designed for full-length CDSs. When applied to ESTs it is imperative that both strands are analysed, as both 5' and 3' ESTs are generated. Therefore the reverse complement of each nucleotide consensus was also analysed. DECODER_default (1) considers only the prediction from the forward strand, whilst DECODER_best (2) uses the more accurate prediction. ESTScan (3) considers both strands of the nucleotide sequence, and was run as a stand-alone process with default settings.

Two arrangements of components within prot4EST were tested. prot4EST_ed (4) implements ESTScan before using DECODER on any remaining untranslated sequences. Conversely, prot4EST_de (5) uses DECODER first followed by ESTScan. The DECODER module in prot4EST considers translations on both the foward and reverse strands of the query sequence.

### 1 Data Sets

#### Test EST dataset for translation

We randomly selected 4000 *Caenorhabditis elegans *ESTs from dbEST [30]. To reduce redundancy, the ESTs were clustered using CLOBB [31]. phrap [9] was then used to derive a consensus sequence for each cluster. This resulted in 2899 nucleotide sequences. To ensure that the consensuses corresponded to a coding region, we carried out a BLASTN search for each consensus against the complete *C. elegans *cDNA dataset available from Wormbase (version 117) [32]. Significant matches were found for 2372 consensuses. Finally, this set was used to query the *C. elegans *protein dataset (Wormpep version 117), thus associating each nucleotide sequence with a corresponding reference polypeptide. A final test set of 2316 consensus sequences was produced.

#### Training datasets

##### 1: Caenorhabditis elegans

Both ESTScan and DECODER require prior gene sequence. The *C. elegans *RefSeq collection was obtained, comprising 21033 entries (December 2003; [33]). A Perl script constructed random training sets giving differing totals of coding nucleotides from 10000 to 350000. Four sets were assembled for each level. The build_tables script (part of the ESTScan package) was used to filter out sequences [18].

We used the same training sets to build the codon usage tables required by DECODER. CUSP from EMBOSS [34] was used to build the tables, and a separate Perl script written to convert the output to that required by DECODER. For any given run of prot4EST the ESTScan HMM training set and codon usage table used were derived from the same training set of *C. elegans *cDNAs.

##### 2: Prokaryote genomes

GenBank entries from 167 complete prokaryote genomes were obtained (May 2004). A Perl script was written to extract the CDS entries and construct a RefSeq-style resource for each prokaryote species (available upon request). If a taxon's genome consisted of more than one megaplasmid the sequences were combined. CDS annotation was not available for 11 genomes. We used the CDS collections for the 156 taxa to determine AT content, construct hidden Markov models and codon usage tables.

##### 3: Arabidopsis thaliana

28960 complete CDS entries for *A. thaliana *were obtained from the RefSeq database [35].

##### 4: Spirurida (Nematoda)

We queried GenBank for all complete CDS entries from species in the nematode order Spirurida.

### BLAST databases

SwissProt (release 42.7) and TrEMBL (release 25.7) [14] were combined to give a SwissAll database. To recreate the situation facing neglected genome analysis, the accession numbers for all proteins from species in the nematode orderRhabditida were retrieved from the NEWT taxonomic database [36] and these entries (~23000) were removed from SwissAll.

### 2 Data collection and analysis

#### Comparison of predicted polypeptides to the 'true' polypeptide

We compared each putative polypeptide predicted from the *C. elegans *test dataset to its cognate reference protein using bl2seq from the NCBI distribution. Default parameters were used except for the theoretical database size (-d), set to 130000, the size of SwissProt. The blast reports were parsed using BioPerl modules [37]. Each *C. elegans *reference protein sequence was also compared to itself using bl2seq with default parameters. The raw and bit scores were recorded.

#### Calculation of comparison statistics

The raw and bit scores were normalised for length and against their theoretical maximum using equation 1, where:

BITlocal is the bit score of the local alignment between the predicted polypeptide and its cognate reference protein,

BITmax is the bit score for the alignment between the reference protein and its self,

WPlength is the length of the wormpep protein that is the reference of the nucleotide consensus translated,

ESTlength is the length of the nucleotide consensus that has been translated.


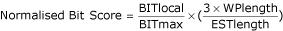


(equation 1)

## Results and discussion

To measure the accuracy of translation two statistics were derived from the comparison of the predicted and reference polypeptides. The **coverage **is the percentage of the predicted polypeptide that aligns with the reference. The **bit score **represents the total of the alignment's pair-wise scores, normalised with respect to the substitution matrix used to calculate these scores. In this study the bit score was itself normalised to compensate for EST length and the maximum possible bit score for each comparison (see Methods, equation 1). The number of consensuses translated that had a significant match to their cognate reference *C. elegans *protein was also recorded for each run.

### The influence of number of training codons

**Figure 3:**
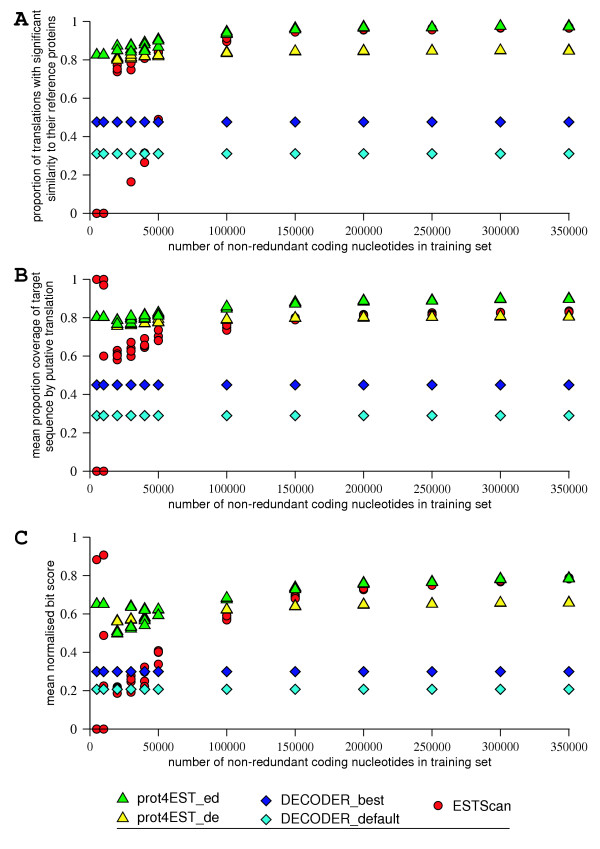
Performance of polypeptide prediction methods under different training regimes. Predicted polypeptides were compared to their reference. Four independent replicates of each training set size were used. a) Proportion of predicted polypeptide peptides having a significant BLASTP match to their reference protein. b) The mean proportion of each sequence covered by a predicted polypeptide. c) The mean relative bit score of each predicted polypeptide compared to its reference protein. The scores in b) and c) are the mean of the sequences translated by each method. The high scores shown by ESTScan at 5000 and 10000 non-redundant coding nucleotides is due to the method returning at most one polypeptide out of the 2316 nucleotides provided.

Both variants of DECODER were unable to produce robust translations for over half the nucleotide sequences no matter how many nucleotides were in the training set (Figure 3). As expected, the inclusion of the reverse complement in the DECODER analysis improved its performance. The inability of DECODER to translate more than 50% of the polypeptides can be traced to its core assumptions. One criterion used is the determination of the most likely initiation methionine. While this is almost always present in full length cDNAs (for which it was designed), the occurrence of any ATG codon in EST consensuses is less certain. We noted that DECODER will try any ATG codon to start its prediction, even if this results in a polypeptide of 2 amino acids in length.

The effect of the number of training nucleotides on ESTScan performance is pronounced. For the majority of the replicates, at each training set size the fraction of predictions that have significant matches to their reference sequence was around 75%, but the number of translations dropped significantly below 250000 training nucleotides. However, for 10000 coding nucleotides or less no robust translations are produced. Additionally, there was variance in the performance of ESTScan when there were between 20000 and 50000 training nucleotides. Examination of these training sets showed no difference in AT content compared to larger training sets, but did suggest that fluctuations in codon usage bias might be involved. The replicates that performed less well comprised sequences with shorter mean length, and had codon biases that were at the extremes of the distribution (not shown). This variation in sequence composition clearly has an effect on the probabilities that populate the HMM used by ESTScan. We suspect that the ability of ESTScan to predict robust translations when trained by datasets of 150000 to 200000 coding nucleotides is inflated as a consequence of the random selection of the training set from the complete *C. elegans *transcriptome. In a genuine situation, when only a small number of full-length CDS exist in the public databases, a significant number will be from highly expressed genes with atypical codon bias and structure. This bias will be evident in real-world CDS sets with fewer than 200 members (150000–200000 coding nucleotides).

When the training sets contained a large number of non-redundant coding nucleotides (> 150000), prot4EST_ed and ESTScan performed equally well (Figure 3a). When the number of coding nucleotides available for training and codon bias determination were reduced, prot4EST translations still showed significant similarity to the correct protein in at least 80% of instances.

The translations produced by prot4EST_ed were the most robust across all totals of coding nucleotides, for both coverage and bit score (Figures 3b & 3c). As the number of coding nucleotides used in training decreased, both measures showed slight reductions.

### Performance of alternative prot4EST architectures

prot4EST_ed produced more robust translations for higher numbers of training sequences. However when smaller totals of training nucleotides were used the translations produced by the alternative architecture, prot4EST_de, were slightly better (Figure 3c), although a smaller proportion of translations were produced with this setup (Figure 3a).

**Figure 4:**
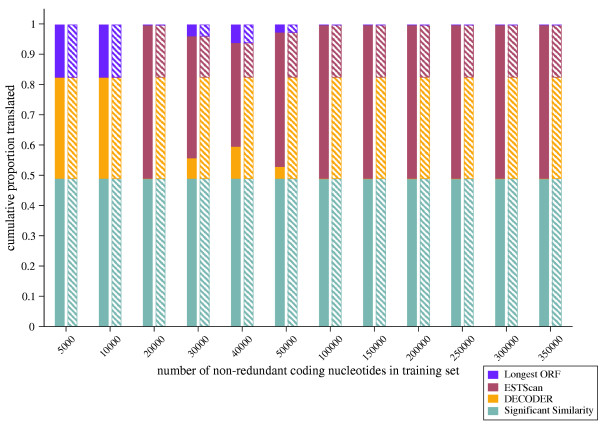
**The relative efficiency of different organisations of DECODER and ESTScan in the prot4EST pipeline. **The proportion of consensus sequences translated by each part of the pipeline for each level of training is shown. bold bars: prot4EST_ed – ESTScan translations were considered before those from DECODER. hashed bars: prot4EST_de – Robust DECODER translations were used in preference to those from ESTScan.

The better performance of prot4EST_ed was examined by following the fate of individual test sequences through the prot4EST pipeline. By employing ESTScan before DECODER, larger training sets allowed the deployment of well trained HMMs (Figure 4). All predictions satisfied length and quality filters, and so were accepted as robust. The corresponding DECODER predictions, while satisfying length filters, were not as robust. As the training sets decreased in size, the ESTScan predictions failed the filters and so were ignored, and DECODER used instead.

### Performance of similarity search

**Figure 5:**
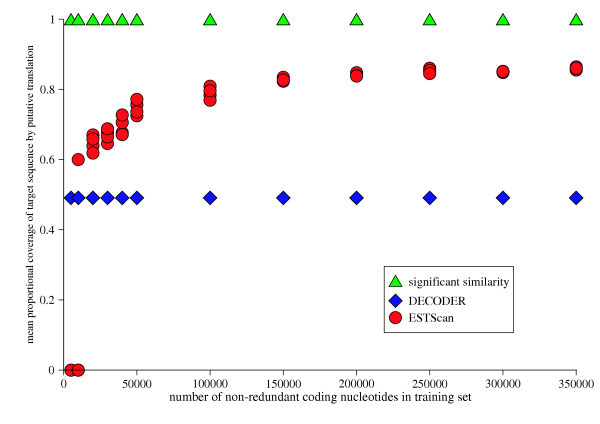
Comparison of HSP tiling, ESTScan and DECODER performance in translating the 1131 consensuses that prot4EST translated using similarity criteria.

Seven sequences out of 2316 were identified as rRNA in tier 1. Tiers 2 and 3 of the prot4EST pipeline exploit any significant sequence similarity between the query sequence and known proteins for coding region determination. This approach identified coding regions from just under half of the consensuses, 1131. Nineteen were identified as mitochondrial genome derived. To benchmark the similarity approach against the other probabilistic methods, the accuracy of predictions from 1131 consensuses were compared. Translations derived from prot4EST tiers 2 and 3 were more robust than those from ESTScan or DECODER (Figure 5).

Given that an increase in the number of non-redundant coding nucleotides used to train ESTScan produces more robust translations, we attempted to use coding regions determined thus far to create larger training sets, with the expectation of improved translations. The results from the BLASTX search against the SwissAll database were checked for matches where the alignment included the start of the protein sequence. These results contained the information required to construct pseudo-CDS entries which can be added to the training set for populating the HMMs of ESTScan. In this study there were only six BLASTX alignments that provided suitable pseudo-CDS, failing to provide any significant increase in the level of non-redundant coding nucleotides. However other species we study have produced higher numbers of pseudo-CDS which prot4EST uses to give improved translations (data not shown).

### Effect of training set and target set sequence composition

As a significant proportion of any EST set will not share similarity with known sequences, *de novo *translation methods need to be trained to as high a level as possible. The question is how this should be done, given the paucity of prior sequence data for individual species. Should CDS from species considered phylogenetically related be combined or should a large set from a model organism be used? A recent study of gene finding in novel genomes has shown a significant effect of sequence composition upon gene structure prediction, with more closely related model genomes providing poor training if the codon bias differs significantly from the genome of interest [25]. The performance of ESTScan was affected by even slight fluctuations in sequence composition. We examined the effect of AT content on the accuracy of translation. The complete CDS complements of 156 prokaryotes were assembled as described in the Methods. This gave a range of AT contents from 28% (*Streptomyces coelicolor*) to 78% (*Wigglesworthia glossinidia*), independent of any bias due the organisms' relatedness to *C. elegans*. The lowest number of non-redundant coding nucleotides was 461,299, in excess of the minimum number suggested for robust training. To explore datasets from more closely related sources all available CDS entries for the nematode order Spirurida (last common ancestor with *C. elegans *was 475–500 MYA [38]), and the plant *Arabidopsis thaliana *[39] were obtained.

**Figure 6:**
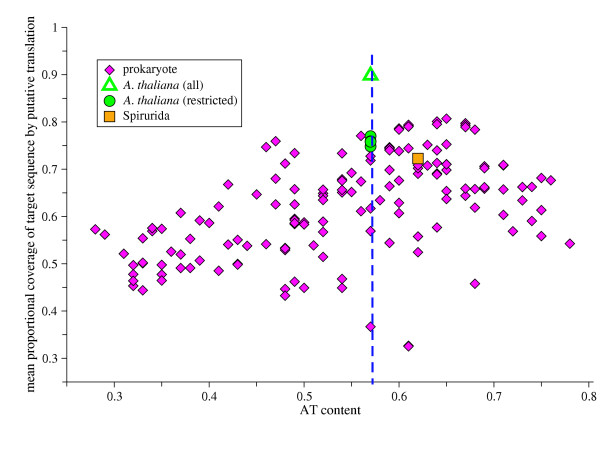
**Effect of AT content of training set upon translation accuracy. **Each purple diamond represents a complete CDS set from a prokaryote genome. The orange box represents all CDS available from the nematode order Spirurida (~230000 non-redundant coding nucleotides). The green triangle represents the complete *Arabidopsis thaliana *RefSeq collection (~30000000 non-redundant coding nucleotides). The green circles are training sets of *A. thaliana *CDS RefSeq entries randomly selected to total ~230000 non-redundant coding nucleotides. The AT content of *C. elegans *is shown by the vertical dashed line.

There was a significant correlation between AT content of the training set and the coverage by the putative polypeptides of their reference *C. elegans *proteins (r = 0.49 P > 0.001) (Figure 6). The most robust predictions were produced by HMMs trained on datasets with an AT content similar to that of *C. elegans*. For the prokaryote training sets, the number of nucleotides used had no significant effect upon performance (data not shown). We note that some prokaryote training sets with AT contents close to *C. elegans *performed poorly: homogeneity of AT content is thus not a panacea. The best performance was obtained using the *A. thaliana *training set, with significantly better coverage than achieved with the more closely related Spirurida. As the plant dataset contained 130 times as many coding nucleotides as did the Spirurida training set, four random *A. thaliana *training sets of comparable size to the Spirurida were built. These smaller training sets still performed better than the Spirurida training set, though not as well as the full CDS collection.

## Conclusions

prot4EST is a protein translation pipeline that utilises the advantages of a number of publicly available tools. We have shown that it produces significantly more robust translations than single methods for species with little or no prior sequence data. Around three quarters of current EST projects are associated with training sets of < 50000 coding nucleotides (Figure 1). Thus prot4EST offers significant improvement in this real world situation. Even with substantial numbers of coding nucleotides, the use of similarity searches means prot4EST is able to outperform the best *de novo *methods. Given the increase in protein sequences submitted to SwissProt/TrEMBL, prot4EST's ability and accuracy can only increase over time. These more accurate translations provide the platform for more rigorous down-stream annotation. Currently we are using the prot4EST pipeline to translate ~95000 nematode consensus sequences from 30 species. These translations will then be passed onto other tools we are developing for EST analysis and annotation (see ).

## Availability and requirements

**Project name: **prot4EST

**Project home page: **

**Operating system(s): **Fully tested on Linux – Redhat9.0, Fedora2.0.

**Programming language: **Perl

Other requirements:

ESTScan2.0 

DECODER rgscerg@gsc.riken.go.jp

BioPerl 1.4 

Transeq 

**License: **GNU GPL

**Any restrictions to use by non-academics: **None for prot4EST source code. DECODER requires a license. See User Guide.

## Authors' contributions

JW performed all the analyses and wrote all the Perl code. MB oversaw the project and suggested additional features.

Both authors shared responsibility for writing this manuscript.

## Figures and Tables

**Table 1:** Description of databases used for similarity searches.

***Source***	Tier*	***Database***	***Link***
ribosomal RNA	1	RBP II	
mitochondrial proteins	2	NCBI	
protein sequences	3	SwissProt/TrEMBL	

*the stage in which the database is used in prot4EST pipeline (see Figure 2).
